# 572. The Burden of RSV Infections Among Nursing Home Residents, Monroe County, NY, October 2022 to April 2023

**DOI:** 10.1093/ofid/ofae631.173

**Published:** 2025-01-29

**Authors:** Kevin Popham, Katherine St George, Christina B Felsen, Ghinwa Dumyati, Brenda L Tesini

**Affiliations:** Universirty of Rochester, Rochester, NY; University of Rochester School of Medicine and Dentistry, Penfield, New York; University of Rochester, Rochester, NY; New York Emerging Infections Program and University of Rochester Medical Center, Rochester, New York; University of Rochester, Rochester, NY

## Abstract

**Background:**

RSV causes significant morbidity and mortality in older adults. Outbreaks are common in nursing home (NH) residents, but little is known about the burden of disease in this vulnerable population. We analyzed population-based data on laboratory-confirmed RSV infections and hospitalizations from the CDC Respiratory Syncytial Virus Hospitalization Surveillance Network to understand the burden of RSV on the NH population compared to community-dwelling older adults.

Weekly Number of Positive RSV Tests Reported by Nursing Homes in Monroe County, NY, October 2022 to April 2023

**Figure:**
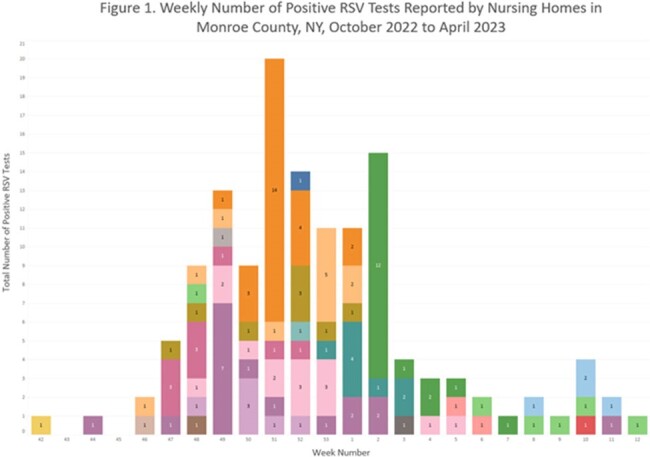

**Methods:**

We reviewed positive RSV tests from all clinical labs utilized by Monroe County, NY residents from October 2022 to April 2023. Cases in patients ≥65 years of age were attributed to a NH or the community through medical chart review. RSV rates were calculated in NH residents using occupied bed census averaged over the season and in community-dwelling residents using county population data. Incidence and outcomes including hospitalization, ICU admission and in-hospital mortality were calculated weekly and across the season.

Healthcare utilization among nursing home residents with laboratory-confirmed RSV hospitalizations in Monroe County, NY, October 2022 to April 2023

**Figure:**
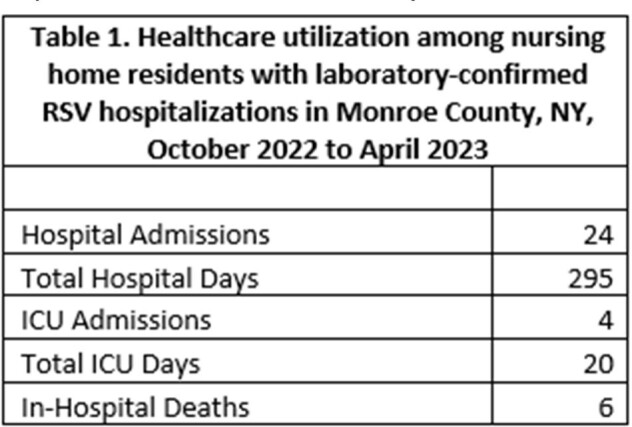

**Results:**

Of 766 laboratory-confirmed RSV cases in patients ≥65 years of age, 134 were among NH residents. Incidence per NH ranged from 25.5 to 3,163.1 per 10,000 residents. NH cases often clustered in time (Figure 1), with a peak weekly attack rate of 17.7% in one facility. Three NHs had a seasonal attack rate >20%.

Of 170 hospitalizations in patients ≥65 years, 24 occurred in NH residents; 18% (24/134) of all NH residents with RSV were hospitalized. NH residents accounted for 295 hospital days (Table 1). Four NH residents required ICU admission, utilizing 20 ICU days, and 6 died in the hospital. NH residents were hospitalized at a rate 5.6 times greater than community dwelling older adults (Table 2).

Rates of laboratory-confirmed RSV infection and hospitalization among adults ≥65 years of age in Monroe County, NY, October 2022 to April 2023

**Figure:**
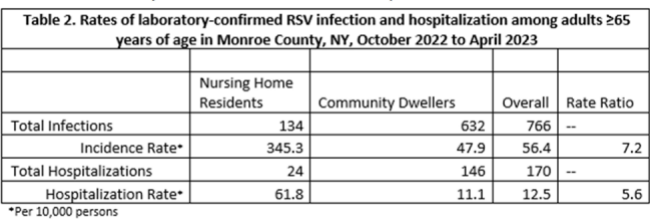

**Conclusion:**

Our data emphasize that NH residents are particularly vulnerable to RSV infection and complications. The tight clustering of laboratory-confirmed cases within facilities likely represents within-facility transmission, the magnitude of which may be underestimated due to NH testing practices. Early recognition of infection and swift implementation of appropriate infection control precautions are needed to limit outbreaks. Additional preventive measures including resident vaccination and policies discouraging working or visiting while ill should be prioritized.

**Disclosures:**

**Brenda L. Tesini, MD**, Merck: Honoraria

